# CD8^+^ T Cells in Atherosclerosis

**DOI:** 10.3390/cells10010037

**Published:** 2020-12-29

**Authors:** Sarah Schäfer, Alma Zernecke

**Affiliations:** Institute of Experimental Biomedicine, University Hospital Würzburg, 97080 Würzburg, Germany; Schaefer_S7@ukw.de

**Keywords:** atherosclerosis, CD8^+^ T cells, inflammation, cytotoxic T cells, single cell RNA sequencing, checkpoint inhibitors, immunotherapy

## Abstract

Atherosclerotic lesions are populated by cells of the innate and adaptive immune system, including CD8^+^ T cells. The CD8^+^ T cell infiltrate has recently been characterized in mouse and human atherosclerosis and revealed activated, cytotoxic, and possibly dysfunctional and exhausted cell phenotypes. In mouse models of atherosclerosis, antibody-mediated depletion of CD8^+^ T cells ameliorates atherosclerosis. CD8^+^ T cells control monopoiesis and macrophage accumulation in early atherosclerosis. In addition, CD8^+^ T cells exert cytotoxic functions in atherosclerotic plaques and contribute to macrophage cell death and necrotic core formation. CD8^+^ T cell activation may be antigen-specific, and epitopes of atherosclerosis-relevant antigens may be targets of CD8^+^ T cells and their cytotoxic activity. CD8^+^ T cell functions are tightly controlled by costimulatory and coinhibitory immune checkpoints. Subsets of regulatory CD25^+^CD8^+^ T cells with immunosuppressive functions can inhibit atherosclerosis. Importantly, local cytotoxic CD8^+^ T cell responses may trigger endothelial damage and plaque erosion in acute coronary syndromes. Understanding the complex role of CD8^+^ T cells in atherosclerosis may pave the way for defining novel treatment approaches in atherosclerosis. In this review article, we discuss these aspects, highlighting the emerging and critical role of CD8^+^ T cells in atherosclerosis.

## 1. Introduction

Cardiovascular diseases are responsible for about 50% of all deaths in Western societies [1]. Atherosclerosis is the predominant underlying cause of heart attack and stroke and can be considered a chronic inflammatory disease of the vessel wall with immune cells accumulating in the intima of large arteries [1,2,3]. Initiated by elevated blood cholesterol levels and lipid deposition in the intima, specialized aortic intima resident macrophages are the first cells to take up these lipids and become plaque foam cells [4]. Concomitant with lipid depositions and its modifications that instigates vascular inflammation at sites of disturbed blood flow, monocytes are in addition recruited to the intima, a mechanism of particular importance during early lesion formation, and differentiate into macrophage foam cells. These lesional macrophages proliferate in the atherosclerotic plaque, a process that contributes to lesion growth [5,6,7]. Beyond macrophages, other immune cells such as T and B cells accumulate within atherosclerotic lesions in large numbers [2]. Recent advances in single cell RNA sequencing have defined the different myeloid and lymphoid cell populations present in the healthy and inflamed vessel wall in atherosclerosis [4,8,9]. In addition, non-leukocyte populations, such as smooth muscle cells (SMC) that can give rise to phenotypically altered macrophage-like cells or fibromyocytes, and mesenchymal cells are emerging as important players in vascular inflammation [10,11,12,13].

Innate and adaptive immune mechanisms play an important and complex role in atherosclerosis. In particular, CD4^+^ T cells and their pro- and anti-inflammatory T helper cell subsets have been shown to modulate the pathogenesis of atherosclerosis, as reviewed elsewhere [2,14]. In addition, CD8^+^ T cells are emerging as important players in atherosclerosis, as shown in mouse models of atherosclerosis and recent clinical studies. This review will provide an overview of the current knowledge on this immune cell subset and their functions in atherosclerosis.

## 2. CD8^+^ T Cells

CD8^+^ T cells are important players in innate and adaptive immune defense mechanisms protecting against extrinsic (e.g., pathogens, viruses and bacteria) but also intrinsic danger (e.g., malignant cells) [15]. Pathogen antigens can be detected by different receptors, including Toll-like receptors (TLRs) [16], and several cytokines with pleiotropic functions are produced by host cells upon infection that activate the adaptive immune system [17]. As part of the innate immune response, CD8^+^ T cells can control infections but also tumors by cross-reacting to self-peptides and reacting to cytokines such as interleukin (IL)-2, IL-12, IL-15 and IL-18 that promote CD8^+^ T cell responses and interferon (IFN)-γ secretion [18].

In adaptive immune responses, naïve CD8^+^ T cells initially interact via the T cell receptor (TCR) with a specific antigen presented through the major histocompatibility complex class I (MHCI; or human leukocyte antigen (HLA)) by antigen-presenting cells (APC). CD8^+^ T cells respond by activation and differentiation into effector T cells, and clonal expansion. This activation and expansion of T cells is tightly regulated to ensure an efficient response to infection while avoiding immunopathology [19]. However, CD8^+^ T cells can also contribute to an excessive immune response, leading to pathological immune-mediated damage.

There are three main functions of cytotoxic T lymphocytes based on MHCI/peptide complex recognition. First, CD8^+^ T cell effector cells secrete cytokines, primarily tumor necrosis factor (TNF)-α and IFN-γ, to induce apoptosis and inflammation. Both of these inflammatory cytokines have antitumor and antiviral effects [20,21,22,23]. Second, cytotoxic T lymphocytes express Fas-ligand (FasL), a T-cell receptor costimulatory molecule. Binding of FasL to its receptor Fas on a target cell results in the downstream activation of caspase signaling cascades to induce apoptosis of the target cell [24,25]. Third, effector CD8^+^ T cells secrete cytotoxic granules, in particular perforin and granzymes, inducing lysis of target cells [26]. After pathogen clearance, effector T cells undergo apoptosis, or differentiate into memory T cells [27]. Terminal differentiation or strong or chronic antigen stimulation can also drive T cells to an exhausted, hypofunctional phenotype [28]. Exhausted CD8^+^ T cells are characterized by a decreased antigen-driven secretion of effector cytokines and elevated expression of inhibitory cell surface receptors, including programmed cell death protein-1 (PD-1) [29,30].

While a predominance of effector and memory T cells is observed after antigenic challenge following the acute phase of inflammation, there are also several CD8^+^ T cell subsets with different function and localization that are less well understood, such as CD8^+^CD25^+^ T cells that constitute a small subset with immune suppressive function [31].

## 3. CD8^+^ T Cells in the Inflamed Artery Wall in Atherosclerosis

The presence of T cells was first evaluated in human atherosclerosis by immunohistochemistry. Carotid artery plaques from atherosclerotic patients recovered after surgery were characterized by immunostaining, and cell types such as macrophages, smooth muscle cells and T lymphocytes, including CD8^+^ T cells, were found within lesions. T cells were, in particular, of the memory T cell phenotype expressing CD45RO and integrin very late antigen-1 [32,33]. The presence of CD8^+^ T cells was also noted within atherosclerotic plaques in mouse models of atherosclerosis [34,35,36].

That CD8^+^ T cells constitute a variable but substantial proportion of the inflamed cellular plaque infiltrate in murine and human atherosclerosis was confirmed by recent single-cell RNA sequencing (scRNA-seq) and cytometry by time of flight (CyTOF) approaches [4,8,9,37,38,39]. These demonstrated that CD8^+^ T cells were almost exclusively found in the atherosclerotic aorta but not in chow-fed control mice [8]. In a meta-analysis of T cells integrating 9 different murine data sets [4], a CD8^+^ T cell cluster was identified by the expression of *Cd8b*, *Cd8a*, and the transcription factor *Eomes*. In addition, CD4^+^CD8^+^ T cells expressing the transcription factors *Tox* and *Sox4* were identified, which may contain an immature T cell population. As expected, aortic CD8^+^ T cells also expressed granzymes and killer cell lectin-like receptors in line with cytotoxic functions [4,8,9].

In human atherosclerotic lesions from carotid arteries, CyTOF analyses identified almost 65% of lesional immune cells as CD4^+^ and CD8^+^ T cells. Remarkably, CD8^+^ T cells were present at higher frequencies in these human atherosclerotic lesions compared to CD4^+^ T cells, and CD8^+^ T cells represented the most enriched cell population compared to their proportions in blood. In contrast, increased frequencies of CD4^+^ CD8^+^ T cell levels were found in blood [39]. Among plaque CD8^+^ T cells, several subclusters were identified by CyTOF. The CD8^+^ T cell compartment comprised 6 clusters, including 1 naïve, 3 effector memory and 2 terminally differentiated effector memory T cell subsets. Among the effector memory cells, one cluster of CD103^+^ CD8^+^ T cells was identified, corresponding to a classical tissue-resident memory T cell subset [39]. These plaque CD8^+^ T cells were more activated than their blood counterparts and showed a heterogeneous spectrum of activation compared to blood. Moreover, CD8^+^ T cell activation and differentiation correlated with TCR clonality in tissue. Notably, CD8^+^ T cells showed an activated phenotype in asymptomatic patients, whereas two clusters of lesional effector memory cells exhibited signs of T cell exhaustion in symptomatic patients, as suggested by expression of programmed cell death protein-1 (PD-1) and lower levels of perforin [39]. CITE-seq and gene expression analyses confirmed that plaque T cells display transcriptional signatures associated with T cell activation, cytotoxicity and exhaustion [39]. Overall, these findings demonstrate that CD8^+^ T cells display a quiescent phenotype in blood, while they display distinct degrees of activation within lesions, and in symptomatic plaques from patients with stroke some CD8^+^ T cells show signs of exhaustion, suggesting the progressive loss of T cell functions in response to chronic persistent inflammation [39]. In another study, a clear exhausted T cell phenotype was not identified in CD8^+^ T cells from atherosclerotic carotid endarterectomy specimen, analyzed by scRNA-seq [38]. Rather, three distinct CD8^+^ T cell subpopulations with an effector-memory phenotype, a terminally differentiated cytotoxic T cell profile and a quiescent, central-memory phenotype were recovered [38]. Interestingly, the T cell cluster expressing CD69, suggestive of recent TCR activation, showed reduced cytotoxic potential, so that it was speculated that not cytotoxic CD8^+^ T cells but more quiescent CD8^+^ T cells subsets react to plaque-specific antigens [38]. The commonly observed relatively low abundance of CD8^+^ T cells with strong cytotoxic gene expression [38,39] raises the question as to the contribution of cytotoxicity to the pathogenesis of human atherosclerosis.

A microenvironment-specific dysfunctional phenotype of CD8^+^ T cells was also observed in advanced aortic lesions of *Apoe^−/−^* mice and human endarterectomy samples by flow cytometric analyses, as evidenced by a decreased expression of TNF-α and IFN-γ as well as increased expression of CD39 by aortic CD8^+^ T cells compared with their counterparts in the spleen. CD39 is a cell surface enzyme catalyzing the hydrolysis of extracellular ATP into ADP, which can be further converted into immunomodulatory adenosine. TCR signaling induces CD39 expression on lesional CD8^+^ T cells. Within lesions, a higher IFN-γ production was observed by CD8^+^ CD39^+^ T cells compared to CD39^−^ T cells, and inhibition of CD39 in mice with advanced atherosclerotic lesions increased IFN-γ production by lesional CD39^+^ and CD39^−^ CD8^+^ T cells in *Apoe^−/−^* mice in an adenosine dependent manner [40]. This suggests that adenosine production induced by CD39^+^ CD8^+^ T cells may regulate lesional IFN-γ production in both a paracrine and autocrine fashion. Continuous TCR signaling in the atherosclerotic plaque microenvironment may thus induce a CD8^+^ T cell phenotype associated with increased CD39 expression that decreases proinflammatory cytokine production. Effects on lesion size or composition, however, were not analyzed in this study [40]. Complete knockout of CD39 in *Apoe^−/−^* mice protects from atherosclerosis, suggesting an overall atherogenic role of CD39 [41]. In these *Cd39*^−/−^ *Apoe^−/−^* mice, effects on other cell populations were noted, including an enhanced cholesterol efflux in macrophages, an increase in plasma HDL levels, as well as an impaired platelet activation, demonstrating a range of complex functions of this enzyme and purinergic signaling in atherosclerosis [41]. Further research investigating the cell-specific effects of CD39 in atherosclerosis are thus warranted.

In another study, a pronounced increase in nonatherogenic RORγt^+^ IL-17-producing CD8^+^ T cells (Tc17) was noted in advanced atherosclerotic lesions of *Apoe^−/−^* mice compared to the spleen [42], which would also be in line with a loss in a proinflammatory and IFN-γ producing cell phenotype in late atherosclerosis.

Little is known about how CD8^+^ T cells migrate to lesion sites. In vitro, an invasion assay demonstrated cytotoxic lymphocyte migration into advanced atherosclerotic lesions, whereas migration into early lesions required additional T cell activation [43], suggesting T-cell receptor activation and/or proinflammatory cytokines as drivers of CD8^+^ T cell recruitment. A number of chemokines/receptors have been associated with the accumulation of T cells and in particular CD4^+^ T cells in atherosclerosis, such as chemokine (CC-motif) receptor 5 (CCR5) and chemokine (CXC-motif) receptor 6 (CXCR6) (as reviewed in [44,45]). However, less is known about how CD8^+^ T cells migrate to the lesion sites. A CD8^+^ T cell population expressing the endothelium homing receptor CX3CR1 was recently described in humans [46,47] that may be directed by CX3CL1-expressing endothelial cells to the inflamed vessel wall [47].

## 4. The Total Pool of CD8^+^ T Cells Aggravates Atherosclerotic Lesion Formation

Mouse models widely used to study the pathogenesis of atherosclerosis have been applied to evaluate the role of CD8^+^ T cells in atherosclerosis. High fat diet feeding over 4 weeks in *Apoe^−/−^* mice induces CD8^+^ rather than CD4^+^ T cell activation and cytokine production in the spleen, which suggested that CD8^+^ T cell activation predominates early immune responses to hypercholesterolemia [48]. However, first analyses using *Apoe^−/−^ Cd8^−/−^* mice did not show a contribution of CD8^+^ T cells to the development of atherosclerosis and no changes in plaque size compared to *Apoe^−/−^* control mice fed a normal chow for 18 weeks or at 1 year of age [34]. Compensatory mechanisms associated with the loss of CD8^+^ T cells, however, were not addressed in this study. Recently, low density lipoprotein receptor-deficient (*Ldlr^−^*^/*−*^) *Cd8^−^*^/*−*^ mice were adoptively transferred with in vitro activated, unpolarized (Tc0) CD8^+^ T cells or IL-17^+^ CD8^+^ T cells (Tc17 CD8^+^ T cells) and fed a Western-type diet. Whereas the transfer of Tc0 CD8^+^ T cells increased atherosclerosis in the aortic root in this model, the transfer of Tc17 cells did not promote atherosclerosis, suggesting that activated Tc0 but not cTc17 cells are atherogenic [42].

In a different approach, an antibody depletion strategy targeting CD8^+^ T cells was employed. Although neutralizing antibodies against the injected antibody were generated, CD8^+^ T cells could be efficiently depleted in atherosclerosis-prone *Apoe^−/−^* or *Ldlr^−/−^* mice by this approach, and the application of an antibody targeting CD8α in *Apoe^−/−^* and *Ldlr^−^*^/*−*^ mice [35,36] as well as application of an antibody directed against CD8β in *Ldlr^−^*^/*−*^ mice [36] led to profound reductions in atherosclerotic lesion burden after 6 or 8 weeks of high-fat diet (HFD) feeding. In both of these models, CD4^+^ T cell activation or polarization were not affected by CD8^+^ T cell depletion [35,36].

## 5. CD8^+^ T Cells Control Macrophage Accumulation and Monocyte Production

The reduction in atherosclerotic lesion formation in both *Apoe^−/−^* and *Ldlr^−^*^/*−*^ mice depleted of CD8^+^ T cells was accompanied by a decrease in the accumulation of plaque macrophages [35,36]. Both CD8^+^ T cell depletion studies demonstrated lower plasma levels of chemokine (CC-motif) ligand 2 (CCL2), which is important for monocyte mobilization from the bone marrow and infiltration of lesions [35,36]. In vitro, neutralization of IFN-γ resulted in a decreased CCL2 production in macrophages cocultured with activated CD8^+^ T cells [36], indicating that CD8^+^ T cells can trigger CCL2 expression in macrophages in an IFN-γ dependent manner. Reduced CCL2 expression in atherosclerotic vessels from anti–CD8-treated *Ldlr*^−/−^ mice could thus have contributed to reduced monocyte recruitment to early lesions. Transfer studies of CD45^+^ cells into control or CD8-depleted *Ldlr^−/−^* mice, however, displayed similar levels of recruited monocytes in the vessel wall, suggesting that CD8^+^ T cells do not directly regulate monocytes migration into atherosclerotic lesions during early atherosclerotic lesion formation [36]. Monocyte production in the bone marrow was lowered after CD8^+^ T cell depletion, accompanied by reduced monocyte counts in the blood circulation, which suggested that CD8^+^ T cells control monopoiesis. Indeed, CD8^+^ T cell depletion reduced levels of granulocyte-monocyte progenitors in the bone marrow, suggesting that CD8^+^ T cells control monocyte expansion by promoting the differentiation of common myeloid to granulocyte-monocyte progenitors and/or through promotion of granulocyte-monocyte progenitor proliferation [36]. The underlying mechanism of CD8^+^ T cell-induced monopoiesis are still unclear. Studies in viral infection reported a similar function of CD8^+^ T cells in modulating monocyte differentiation, and here CD8^+^ T cell-secreted IFN-γ induced IL-6 secretion in mesenchymal stromal cells to increase monocyte production [49]. IFN-γ blockade in high fat diet fed *Ldlr^−/−^* mice in part recapitulated the effects of CD8^+^ T cell depletion on bone marrow monopoiesis during hypercholesterolemia in *Ldlr*^−/−^ mice [36], suggesting that IFN-γ secretion by CD8^+^ T cells may at least in part contribute to monopoiesis in the bone marrow and in consequence contribute to Ly6C^hi^ monocytosis during atherogenesis. Reduced monocyte levels in blood could then lead to reduced monocyte recruitment, resulting in lower macrophage accumulation in CD8^+^ T cell-depleted atherosclerotic mice [36,50].

## 6. Cytotoxic Functions of CD8^+^ T Cells

**Figure 1:**
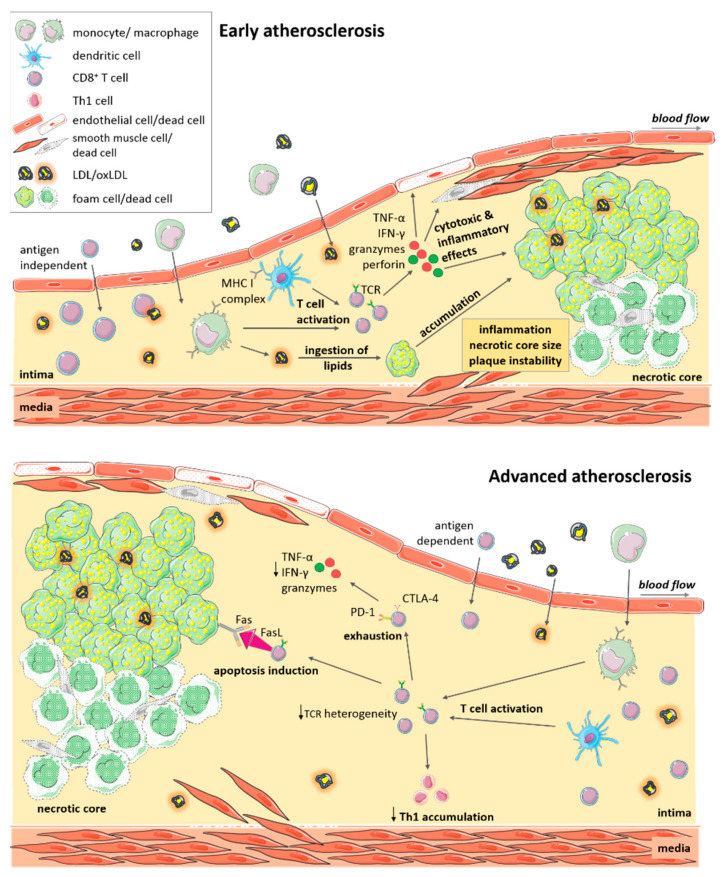
CD8^+^ T cell functions in atherosclerosis. Immune cells including CD8^+^ T cells, infiltrate atherosclerotic lesions. CD8^+^ T cells are activated and expand locally in a potentially antigen-dependent manner. CD8^+^ T cells produce cytokines (TNF-α, IFN-γ) and cytotoxic mediators (granzyme, perforin), promoting plaque inflammation and cytotoxic cell death of macrophages, smooth muscle cells and endothelial cells. These effects promote expansion of the necrotic core, and a more instable plaque phenotype. In advanced atherosclerosis, CD8^+^ T cells are of reduced T-cell receptor (TCR) heterogeneity. Persistent activation may drive T cell exhaustion and reduced cytokine and cytotoxic functions. Activation of the Fas-FasL pathway induces apoptosis of foam cells and decreased Th1 T cell accumulation. Killing of macrophages may lower plaque burden but also increase inflammation in advanced atherosclerosis. Local cytotoxic responses towards endothelial cells may promote plaque erosion at culprit sites of acute coronary syndromes.

In line with cytotoxic function of CD8^+^ T cells, a reduction in the plaque necrotic core area was evidenced upon antibody-mediated CD8^+^ T cell depletion in *Ldlr^−^*^/*−*^ and *Apoe^−/−^* mice [35,36], implying a contribution of CD8^+^ T cells to cell death within lesions. This notion was further corroborated in adoptive transfer experiments in lymphocyte-deficient *ApoE^−/−^Rag-2^−/−^* mice in which CD8^+^ T cells deficient in the cytotoxic molecules perforin and granzyme B, as well as in the cytokine TNF-α but not IFN-γ, failed to increase atherosclerosis as induced by the adoptive transfer of wildtype CD8^+^ T cells. This demonstrates a critical role of cytotoxic functions and TNF-α production by CD8^+^ T cells in lesion development. Vascular inflammation was associated with increased numbers of apoptotic macrophages, endothelial and smooth muscle cells in mice receiving perforin or granzyme-B-competent cells [35], suggesting that CD8^+^ T cell-mediated cytotoxicity contributes to necrotic core formation by inducing lesional cell death of these cell types, and an instable plaque phenotype during lesion development (Figure 1).

Contrasting these findings in early atherosclerosis, the role of CD8^+^ T cells may be more complex in advanced atherosclerosis. When *Ldlr^−/−^* mice were fed a high fat diet for 10 weeks and then in addition received the CD8β depleting or control antibody for another 6 weeks, lesion size was increased, together with an expansion of macrophage and necrotic core area, indicative of a decrease in plaque stability. In this study, it was shown that CD8^+^ T cells promoted FasL-dependent-apoptosis of lipid-laden macrophages, as deduced from increased lesional macrophage content and an accumulation of Tbet^+^ CD4^+^ T cells after CD8^+^ T cell depletion or FasL inhibition. By inducing Fas-FasL-mediated apoptosis of macrophages, lowering macrophage lesion burden, CD8^+^ T cells may thus provide local protection in advanced atherosclerosis (Figure 1) [51]. Another study investigating the role of Casitas B-cell lymphoma-B (CBL-B) in advanced atherosclerotic lesions also noted CD8^+^ T-cell-driven macrophage death in advanced lesions, but this was associated with increased inflammation and lesion burden. CBL-B is an E3 ubiquitin ligase that modulates activation and polarization of T cells. Genetic deletion of CBL-B entailed an increase in plaque T cells in 20 week old *Apoe^−/−^* mice, and especially in cytotoxic T cells that were resistant to apoptosis and regulatory T cell-mediated suppression. *Cblb^−^*^/*−*^*Apoe^−^*^/*−*^ CD8^+^ T cells showed enhanced IFN-γ and granzyme B production and plaque macrophage killing, which resulted in increased lesional inflammation and a significant increase in plaque area [52]. In this study, CD8^+^ T cells thus seemed to promote inflammation, the accumulation of proinflammatory Th1 cells and atherosclerosis [52], similar to findings in early atherosclerosis. In this study, the authors also noted an effect on monocyte influx during initial stages of atherosclerosis [52], so also other cell types may have come into play in this study. More sophisticated mouse models allowing the cell-specific deletion of e.g., cytotoxic mediators, will reveal the exact function of CD8^+^ T cells in atherosclerosis in the future. Moreover, it remains to be defined whether specific antigens presented by plaque cells elicit such cytotoxic CD8^+^ T cell responses.

## 7. Antigen-Specific CD8^+^ T Cell Responses in Atherosclerosis

In comparison to circulating T cells, plaque CD8^+^ T cells are highly activated [38,39,53] raising the question as to the antigen-specificity of this cell subset. It has been shown that the aorta can support T-cell priming of naïve CD4^+^ T cells, and that naïve T cells traffic between the circulation and vessel wall [54]. Whether CD8^+^ T cells can be primed or activated locally within lesions is unclear. Interestingly, several studies suggested local antigen-specific T cell responses within lesions [53,55]. Analyzing the T-cell antigen receptor mRNA in atherosclerotic lesions at different stages of disease development revealed skewing towards a highly restricted T cell receptor repertoire in both fatty streaks and fibrofatty plaques of *Apoe^−/−^* mice. This indicates oligoclonal expansion of T cells [55] and suggests an initial recruitment of an antigen-independent heterogeneous T cell population, followed by selective T cell expansion reactive to local antigens during atheroprogression (Figure 1) [55].

Atherosclerosis-related antigens were detected in human and experimental models of atherosclerosis, including oxidized LDL (oxLDL), apolipoprotein B-100 (ApoB100) and heat shock proteins [56,57,58]. For CD4^+^ T cell related antigen it was already demonstrated that fragments of these peptides are processed by APCs, traffic to their cell surface and are presented via the MHCII complex [56,59]. For CD8^+^ T cells this process has not been investigated. However, in vitro data demonstrate that epitopes of oxLDL (but not native LDL) can also activate CD8^+^ T cells in the presence of dendritic cells and may thus serve as a self-antigen in atherosclerosis [60,61]. Furthermore, a MHCI-specific pentamer including a peptide fragment of ApoB100 demonstrated the existence of antigen-dependent T cells in vivo in 13 week old *Apoe^−/−^* mice compared to healthy wildtype mice [62].

**Figure 2:**
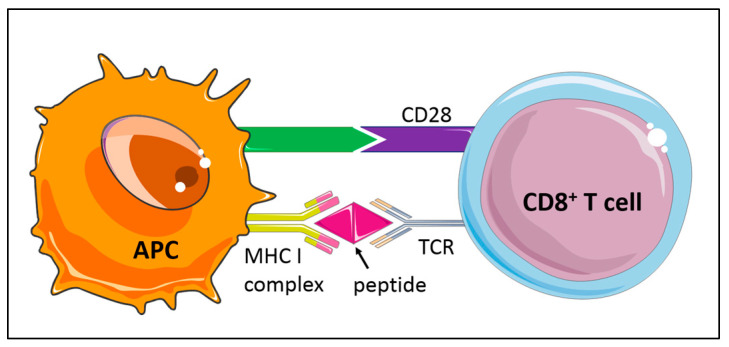
Activation of naïve CD8^+^ T cell subsets is based on the interaction of the T-cell receptor (TCR) with antigen presented on major histocompatibility class I (MHCI) by antigen-presenting cells (APC). Costimulatory or inhibitory signals, such as the CD28/CTLA-4 family of T cell regulators, promote or limit T cell responses.

Antigen-specific CD8^+^ T cell activation requires MHCI-dependent antigen-presentation by APCs (Figure 2). Several studies have explored MHCI-dependent CD8^+^ T cell responses. *C57BL/6* mice deficient for the surface expression of MHCI were created by targeting the β2 microglobulin locus. These MHCI-deficient mice developed increased atherosclerotic fatty streaks in the aorta after feeding a high cholesterol diet for 15 weeks and showed lower levels of CD8^+^ T cells [63], indicating antiatherogenic functions of MHCI-related antigen presentation and activation of CD8^+^ T cells. In another study, *Apoe^−/−^* mice deficient in the transporter associated with antigen processing-1 (TAP-1), which is required for MHC class I antigen presentation and important for thymic differentiation of T cells, displayed dramatically reduced CD8^+^ T cell levels but unaltered atherogenesis [64]. It should be noted, however, that loss of CD8^+^ T cells was compensated by increased CD4^+^ T cell levels and increased CD4^+^ T cell infiltrates in atherosclerotic lesions in this study, which may have masked effects of reduced CD8^+^ T cells on disease progression. Additional studies will be required to further evaluate the physiological occurrence and function of antigen-specific CD8^+^ T cells in atherosclerosis.

## 8. Costimulation/-Inhibition of CD8^+^ T Cells in Atherosclerosis

Antigen-specific T cell responses are modulated by specific receptors bridging CD8^+^ T cells and APCs, serving as costimulatory or inhibitory signals that promote or limit T cell responses, respectively [65,66]. The immune checkpoint programmed cell death protein 1 (PD-1, or CD279) belonging to the extended CD28/CTLA-4 family of T cell regulators serves as a coinhibitory cell surface receptor (Figure 2), essential for T cell tolerance. Deficiency in PD-1 or its ligand PD-L1 in *Ldlr^−/−^* mice has been shown to trigger strong CD4^+^ and CD8^+^ T cell infiltrates in atherosclerotic plaques and a massive lesion growth despite an expansion of atheroprotective regulatory T cells [65,67,68].

In human atherosclerotic plaques, the costimulatory receptor CD137 (4-1BB) was found to be expressed on activated T cells and to act in a TCR-independent manner [69,70]. This receptor belonging to the necrosis factor superfamily member serves as a costimulatory factor for T cells and drives T cell proliferation and cytokine production [69,71]. CD137 ligand (CD137L) is present on the cell surface of several APCs, including B cells, macrophages and dendritic cells, and increases in expression upon activation under chronic inflammatory conditions [70]. Treatment of *Apoe^−/−^* mice with a CD137 agonist caused increased T cell infiltration consisting mostly of CD8^+^ T cells, and elevated levels of proinflammatory cytokines [69]. Similarly, increased CD8^+^ T cell plaque infiltrates were seen in adoptive transfer experiments using wild type but not CD137-deficient CD8^+^ T cells specific for an unrelated antigen (ovalbumin) transferred into proprotein convertase subtilisin/kexin type 9 (PCSK9)-mediated hyperlipidemic mouse models upon ovalbumin immunization [71]. These lesional CD137^+^ effector CD8^+^ T cells promoted infiltration of endogenous CD8^+^ T cells with IFNγ-producing potential, whereas CD137-deficient CD8^+^ T cells exhibited impaired vessel infiltration, minimal IFN-γ production, and lead to also reduced infiltration of endogenous CD8^+^ T cells. CD137 costimulation was thus demonstrated to serve as a stimulus for effector CD8^+^ T-cell infiltration and persistence within atherogenic foci independently of plaque-specific antigen recognition. Together, these data demonstrate that costimulatory factors control the accumulation and activation of CD8^+^ T cells in atherosclerosis, thereby modulating CD8^+^ T cell function in atherosclerosis. Whether such modulation would also relate to cytotoxic functions and antigen-specific effects remains to be uncovered.

## 9. Regulatory CD8^+^ T Cell Subsets

While the majority of studies point toward proatherogenic functions of CD8^+^ T cell activation and cytokine secretion in atherosclerosis, also immunoregulatory CD8^+^ T cell subtypes have been identified. In *Apoe^−/−^* mice, the presence of CD8^+^ CD25^+^ T cells was noted in atherosclerotic lesions, and these cells have immunosuppressive characteristics. In adoptive transfer experiments, CD8^+^CD25^+^ T cells decreased plaque size, reduced macrophage content and inhibited CD4^+^ T cell proliferation, while adoptive transfer of CD8^+^CD25^−^ T cells did not show any effects on atherosclerosis burden [72].

Another subset of regulatory CD8^+^ T cells was uncovered in Qa-1-deficient mice. Qa-1 is the murine homolog of human leukocyte antigen-E (HLA-E), a nonclassical MHC class Ib molecule that can interact with the T cell receptor, leading to activation and expansion of antigen-specific CD8^+^ T cells, but also the CD94/NKG2A receptor on CD8^+^ T cells, natural killer (NK) and NKT cells, attenuating the activities of these cells [73,74,75]. To investigate the consequences of a specific interaction of the Qa-1 molecule with the T cell receptor, a transgenic mouse model carrying a point mutation (D227K) in the Qa-1 molecule was employed. Genetic disruption of CD8^+^ T cell function in this study resulted in an increase in atherosclerosis in *Apoe^−/−^* mice, associated with a decrease in CD8^+^ regulatory T cells, an expansion of T follicular helper and germinal center B cells (GC B cells), increased levels of plasma immunoglobulin levels, and the formation of adventitial ectopic germinal centers in atherothrombotic arteries. When mice were treated with an anti-ICOSL antibody that is known to impair T follicular helper cell development, this restored the control of the T follicular helper cell-germinal center B cell axis and decreased lesion formation. This suggests that Qa-1 restricted regulatory T cells (Tregs) can inhibit the T follicular helper cell-mediated activation of germinal center B cells, which inhibits lesion development [74]. Via this mechanism, CD8^+^ T cells control lesion formation systemically, outside of local effects in the plaque.

## 10. CD8^+^ T Cells and Clinical Manifestations of Atherosclerosis

From the cardiovascular arm of the Malmö Diet and Cancer Study, 700 patients were analyzed for the association of baseline circulating CD8^+^ T cells and carotid artery disease [76]. Interestingly, patients with lower circulating CD8^+^ T cell levels had a reduced risk of myocardial infarction [76], whereas high frequencies of CD8^+^CD25^+^ T cells were associated with an increased, and IFN-γ^+^CD56^−^CD8^+^ T cells with a lower degree of carotid artery stenosis [76]. In other studies, an expansion of CD8^+^ T cells expressing the IL-6 receptor α [77] chain or coexpressing both PD-1 and Tim-3 was described in patients with clinical manifestations of atherosclerosis [78]. This suggests that distinct blood CD8^+^ T cell subsets are associated with atherosclerosis and different pathological outcomes. Whether frequencies of circulating T cell subsets, however, correlate with or predict local plaque CD8^+^ T cell phenotypes or infiltration of CD8^+^ T cells to the vessel wall or other organs, such as lymph nodes or the bone marrow, is still unclear.

In this respect, recent analyses at the single-cell level are of interest, as outlined above. Mass cytometry of blood and carotid artery plaques of cohorts of 15 and 23 patients showed that CD8^+^ T cells were the most enriched cell population compared to blood, and that plaque CD8^+^ T cells (similar to CD4^+^ T cells) displayed an effector memory T cell phenotype. This was also confirmed by cellular indexing of transcriptomes and epitopes by sequencing (CITE-seq) of blood and paired atherosclerotic plaque tissue of one patient, showing higher levels of naïve and central memory T cells in blood, and CD8^+^ T cells in plaque displaying the activation markers HLA-DR and CD38. However, immune cell profiling of circulating T cells in blood and plaque tissue of a larger number of patients will be required to further elucidate whether circulating CD8^+^ T cells could function as biomarkers of atherosclerosis.

Comparing immune cells from 8 asymptomatic and 7 symptomatic patients with recent cardiovascular events by CITE-seq further uncovered an exclusive enrichment of CD127^low^ CD8^+^ T cells in symptomatic plaques [39], corresponding to effector T cells. scRNA-seq analyses furthermore showed a CD8^+^ T cell cluster distinctive in the coactivation of IFN and T cell exhaustion markers in symptomatic patients. This may reflect chronic T cell activation in symptomatic patients. In contrast, CD8^+^ T cell clusters characterized by inflammatory signaling, and pathways related to T cell activation and migration were observed in asymptomatic patients [39].

Using a computational approach to predict cell-to-cell interactions, it was furthermore deduced that T cells contribute to proinflammatory responses and lipid accumulation in macrophages in asymptomatic lesions, whereas in symptomatic patients, T cells may activate proinflammatory signaling in macrophages, e.g., via CCL5-CCR5 interactions [39], a chemokine signaling axis associated with plaque vulnerability [79].

In human atherosclerosis, CD8^+^ T cells are mainly found in the plaque shoulder and region of the fibrous cap [80]. The recent clinical OPTICO-ACS study investigated the microenvironment of atherosclerotic culprit lesions in a cohort of 170 patients with acute coronary syndromes by high resolution optical tomography imaging und further immunological phenotyping. Culprit lesions either showed plaque rupture (RFP-ACS) or had an intact fibrous cap (IFC-ACS) indicative of superficial plaque erosion as a driver of plaque instability. In IFC-ACS lesions, an enrichment in both CD4^+^ and CD8^+^ T cells was observed when compared to RFP-ACS, in parallel with increased levels of cytotoxic effector molecules such as granzyme A, perforin or granulysin. In vitro, CD8^+^ T cells and their cytotoxic effector molecules induced apoptosis of human endothelial cells, and an enhanced adhesion of pre-activated CD8^+^ T cells was observed on endothelial cells under disturbed flow conditions [81]. Although causality remains to be confirmed in future research, this important study suggests that local cytotoxic CD8^+^ T cell responses may trigger endothelial damage and plaque erosion in the presence of local alterations of shear stress [81,82], which could be an important and underappreciated cause of acute coronary syndrome.

Although these exciting findings open up new perspectives on potential functions of CD8^+^ T cells in atherosclerosis, additional studies involving larger patient cohorts, a site-specific interrogation of the T cell phenotypes, and functional investigates will be necessary to further our understanding of the exact roles of CD8^+^ T cells in atherosclerosis and its clinical manifestations.

## 11. CD8^+^ T Cells and Immunotherapy

Vaccination approaches are prominent strategies aiming at the prevention of atherosclerosis. The human ApoB100 peptide already reached preclinical development, but no clinical trial was performed so far [56]. Increased splenic CD8^+^ CD25^+^ IL-10^+^ T cell levels were noted early after immunization with p210, an ApoB100-related peptide vaccine, in atherosclerosis-prone *Apoe^−/−^* mice, accompanied by a reduction in atherosclerotic lesion size. CD8^+^ T cells from p210 immunized mice developed a preferentially higher cytolytic response against p210-loaded dendritic cells in vitro and reduced dendritic cells at the site of immunization and within the plaque [72,83], an effect that was lost after depleting CD25 from CD8^+^ T cells [84]. These data suggest that CD8^+^ CD25^+^ T cells can exert protective effect in atherosclerosis after p210 immunization by reducing immunogenic dendritic cell levels by direct cytotoxic effects, leading to a reduction in lesion formation [83]. Furthermore, vaccination of *Apoe^−/−^* mice with the ApoB100 derived peptides P2 or P45 conjugated to BSA increased the expression of CD8a^+^ CD25^+^ FoxP3^+^ regulatory T cells, accompanied by a significant increase in the secretion of IL-10 with no effects on IFN-γ levels [84]. These studies provide evidence that CD8^+^ T cells are targeted and could be involved in the protective effects of vaccination approaches [56]. However, vaccination with a human HLA-A2 restricted epitopes derived from human ApoB100, which induced ApoB100 specific CD8^+^ T cell responses, did not impact plaque size and cellular composition in HLA-A2 and human ApoB100 transgenic *Ldlr^−^*^/*−*^ mice fed a Western diet for 9 weeks [85]. Furthermore, no recall response could be detected in cultures of cells isolated from the aortic arch in contrast to cells derived from the spleen or lymph nodes, suggesting that the atherosclerotic environment may have impaired CD8^+^ T cell activation and limited the site-specific effects of CD8^+^ T cells [85]. Whether a vaccination strategy aiming at the induction of antigen-specific CD8^+^ T cells may ultimately be beneficial will likely depend on the elucidation of the exact differentiation pathways und antigen-triggered responses of specific CD8^+^ T cell subsets and their effects on lesion formation.

Another potential therapeutic strategy of inducing immunologic tolerance is the application of modified anti-CD3 antibodies. In vitro and in vivo studies demonstrated anti-CD3 antibodies as activators of CD8^+^ T cells and the induction of regulatory CD8^+^CD25^+^ T cells. Patients treated with anti-CD3 showed increased Foxp3 on CD8^+^ T cells suggesting the induction of immune regulation in humans using a week TCR agonist [86]. Furthermore, clinical trials of low-dose IL-2 therapy were shown to reduce atherosclerosis by regulatory T cell-mediated suppression of inflammation. Upon T-cell receptor ligation and costimulation, both naive CD4^+^ and CD8^+^ T cells transiently express CD25 and respond to IL-2 to promote their terminal differentiation. However, low-dose IL-2 therapy did not induce clonal expansion or activation of CD8^+^ effector T cells. Rather, CD4^+^ regulatory T cells were induced that controlled autoreactive CD8^+^ effector T cells [87], an effect that could have contributed to the atheroprotective effects. IL-2 therapy has also been evaluated in a clinical study treating hepatitis C virus (HCV), in which a strong increase in CD25^+^ FoxP3^+^ CD8^+^ Tregs was observed [88], a finding that could warrant further studies also in the context of atherosclerosis. A novel strategy for selective antigen-specific redirection based on a nanoparticle approach could also be of interest. Nanoparticles can be used as ligand-multimerization platform for specific cellular receptor activation on murine and human derived cells. Designing peptide-MHC-nanoparticles with autoimmune-relevant peptide was shown to able to expand regulatory T cells in autoimmunity [89,90]. Triggering antigen-specific regulatory CD8^+^ T cells using this nanoparticle approach could also be tested in atherosclerosis. Advances in our understanding of the exact cell subtypes and their interactions could help to design more precisely tailored cardiovascular immunotherapies.

Immune checkpoint inhibitors are widely used in cancer care, promoting the immune system to identify and target cancer cells. Several human and murine studies already demonstrated accelerated immune processes after blocking or depleting PD-1 or its ligand PD-L1, with parallel increased lesional CD4^+^ and CD8^+^ T cell levels in atherosclerosis [68,91,92]. The combined treatment of mice with immune checkpoint inhibitors targeting both PD-1 and cytotoxic T lymphocyte-associated antigen-4 (CTLA-4), or CTLA inhibition alone, increased both CD4^+^ effector T cell and CD8^+^ cytotoxic T cell numbers in the arterial wall of hyperlipidemic mice [93]. A recent clinical study furthermore demonstrated that cardiovascular events were higher after initiation of immune checkpoint inhibitor treatment, potentially mediated by accelerated progression of atherosclerosis [94]. The exact contribution of CD4^+^ versus CD8^+^ T cells to the proatherogenic effects of immune checkpoint inhibitors is still unclear, but understanding their specific roles in mediating vascular inflammation and injury may help to design strategies to antagonize these effects in patients treated with checkpoint inhibitors in cancer therapy.

## 12. Conclusions

Here, we highlighted recent evidence demonstrating that CD8^+^ T cells are emerging as a critical cell population in atherosclerosis. CD8^+^ T cells can exert both atheroprotective and proatherogenic functions in atherosclerosis, and different T cells subsets exist. CD8^+^ T cells are recruited to the inflamed vessel wall in atherosclerosis [8]. Little is still known about how CD8^+^ T cells migrate to lesion sites, but CX3CR1 was recently uncovered to direct CD8^+^ T cells into lesions in humans [47].

Within plaques, CD8^+^ T cells exhibit varying degrees of activation and cytotoxic functions, and some cells show markers of T cell exhaustion, particularly in symptomatic patients, which may suggest a progressive adaptation and possibly loss in function in response to chronic, persistent inflammation (Figure 1) [39]. In support of this concept, microenvironmental factors within the plaque have been shown to promote a dysfunctional phenotype of CD8^+^ T cells. Related to continuous TCR signaling, increased CD39 expression for instance was shown to induce a CD8^+^ T cell phenotype associated with decreased cytokine production [40]. The exact mechanisms controlling T cell activity within lesions and the cellular crosstalk involved herein, however, are still incompletely understood. Moreover, it is unclear, if a loss in functionality of lesional CD8^+^ T cells affects plaque inflammation.

Several studies have noted an oligoclonal expansion of T cells within lesions, suggesting local expansion. Recent evidence has further provided evidence of antigen-specific CD8^+^ T cell responses in atherosclerosis with oxLDL being able to activate CD8^+^ T cells in vitro [60], and the identification of CD8^+^ T cells reactive to a peptide fragment of ApoB100 in atherosclerotic mice [62]. The study of MHCI-dependent CD8^+^ T cell responses, however, have yielded ambiguous findings, with MHCI-deficient mice developing increased atherosclerosis [63] and *Apoe^−/−^* mice deficient in the transporter associated with antigen processing-1 (TAP-1), which is required for MHC class I antigen presentation, showing reduced CD8^+^ T cell levels but unaltered atherogenesis [64]. In these mice, however, compensatory mechanisms are in place and it is conceivable that these mouse models do not allow teasing out the role of CD8^+^ T cell responses towards specific and atherosclerosis-related antigens.

Globally depleting all CD8^+^ T cells reduces atherosclerotic lesion formation and uncovered a function of CD8^+^ T cells in monocyte production in the bone marrow subsequently controlling plaque macrophage accumulation, as well as a critical functions as cytotoxic cells [35,68]. Cytotoxicity towards antigen-presenting cells [84] may reduce immunostimulatory functions, whereas a reduction in macrophage burden could potentially be protective [51] or promote plaque instability [35,36]. The death of endothelial cells, again, could contribute to plaque erosion and thereby trigger acute thrombo-occlusive events, as suggested by a recent clinical study comparing culprit lesion phenotypes in acute coronary syndromes and the identification of CD8^+^ T cells at this site [81,82].

The abundance and functionality of cytotoxic CD8^+^ T cells at different stages of lesion formation and the varying locations within plaques has not been evaluated in detail, similar to the presence of other (CD8^+^) T cell subsets with potential immunosuppressive function that may antagonize pathogenic CD8^+^ T cell activity. Advancing our insights into the detailed functions of CD8^+^ T cells and the mechanisms controlling their phenotypes in atherosclerosis will be prerequisite to the development of novel therapeutic approaches to specifically target certain CD8^+^ T cell subsets or functions to combat atherosclerosis in the future.

## Data Availability

Not applicable.
